# The HIV-1 envelope cytoplasmic tail protects infected cells from ADCC by downregulating CD4

**DOI:** 10.1128/mbio.01763-25

**Published:** 2025-09-08

**Authors:** Alexandra Tauzin, Étienne Bélanger, Jérémie Prévost, Halima Medjahed, Catherine Bourassa, Frederic Bibollet-Ruche, Jonathan Richard, Beatrice H. Hahn, Andrés Finzi

**Affiliations:** 1 Centre de Recherche du CHUM 177460, Montreal, Québec, Canada; 2 Département de Microbiologie, Infectiologie et Immunologie, Université de Montréal 5622, Montreal, Québec, Canada; 3 Department of Medicine, University of Pennsylvania 183829, Philadelphia, Pennsylvania, USA; 4 Department of Microbiology, University of Pennsylvania 332252, Philadelphia, Pennsylvania, USA; Dana-Farber Cancer Institute, Boston, Massachusetts, USA

**Keywords:** HIV-1, Env, cytoplasmic tail, CD4, ADCC, CD4-induced antibodies

## Abstract

HIV-1-mediated CD4 downregulation is a well-known mechanism that protects infected cells from antibody-dependent cellular cytotoxicity (ADCC). While CD4 downregulation by HIV-1 Nef and Vpu proteins has been extensively studied, the contribution of the HIV-1 envelope glycoprotein (Env) in this mechanism is less understood. While Env is known to retain CD4 in the endoplasmic reticulum (ER) through its CD4-binding site (CD4bs), little is known about the mechanisms underlying this process. Here, we show that the cytoplasmic tail of Env is a major determinant in CD4 downregulation. This function is highly conserved as it was observed with nine different infectious molecular clones from four clades. The small but significant accumulation of CD4 at the surface of cells infected with Env cytoplasmic tail-deleted viruses is sufficient to trigger Env to adopt a more “open” conformation. This prompted recognition of HIV-1-infected cells by plasma from people living with HIV (PLWH) and several families of CD4-induced (CD4i) antibodies, leading to the elimination of these cells by ADCC. While cytoplasmic tail truncations are known to enhance Env expression at the cell surface, this did not fully explain the increased recognition of infected cells by CD4i antibodies and plasma from PLWH. Introduction of the CD4bs D368R mutation, which abrogates CD4 interaction, decreased Env recognition and ADCC. Overall, our results show that CD4 downregulation by the cytoplasmic tail of Env contributes to the protection of infected cells from ADCC.

**IMPORTANCE:**

HIV-1-mediated CD4 downregulation is a central mechanism involved in the protection of infected cells from antibody-dependent cellular cytotoxicity (ADCC). CD4 downregulation prevents the premature interaction between HIV-1 envelope glycoproteins (Env) and CD4, which would otherwise “open” Env and expose vulnerable epitopes recognized by CD4-induced antibodies present in the plasma from people living with HIV. While the mechanisms of CD4 downregulation by the viral accessory proteins Nef and Vpu have been elucidated, the function of Env in this process is less clear. Here, we show that the cytoplasmic tail of Env plays an important role, thus contributing to the protection of infected cells from ADCC.

## OBSERVATION

CD4 downregulation is a central mechanism developed by HIV-1 to protect infected cells from antibody-dependent cellular cytotoxicity (ADCC) (1, 2). If CD4 is not downregulated, it interacts with HIV-1 envelope glycoproteins (Env) exposing otherwise occluded vulnerable epitopes recognized by CD4-induced (CD4i) antibodies present in plasma from people living with HIV (PLWH) (3).

It is well established that HIV-1 uses several proteins to downregulate CD4 from the surface of infected primary CD4^+^ T cells. The accessory protein Nef targets CD4 molecules already present at the plasma membrane by engaging the clathrin-associated adaptor protein 2 (AP-2) complex, which accelerates CD4 endocytosis and degradation by the lysosomes (4–6). The accessory protein Vpu targets newly synthesized CD4 in the endoplasmic reticulum (ER) by recruiting β-TRCP and targeting CD4 to the ER-associated degradation pathway (7, 8). Env has also been described to be involved in CD4 downregulation, although its effect is less marked than for Nef or Vpu (9–11). While it has been shown that Env retains CD4 in the ER and that the CD4-binding site (CD4bs) of Env is required for this retention, little is known about additional determinants of Env that are involved in this process.

**Fig 1:**
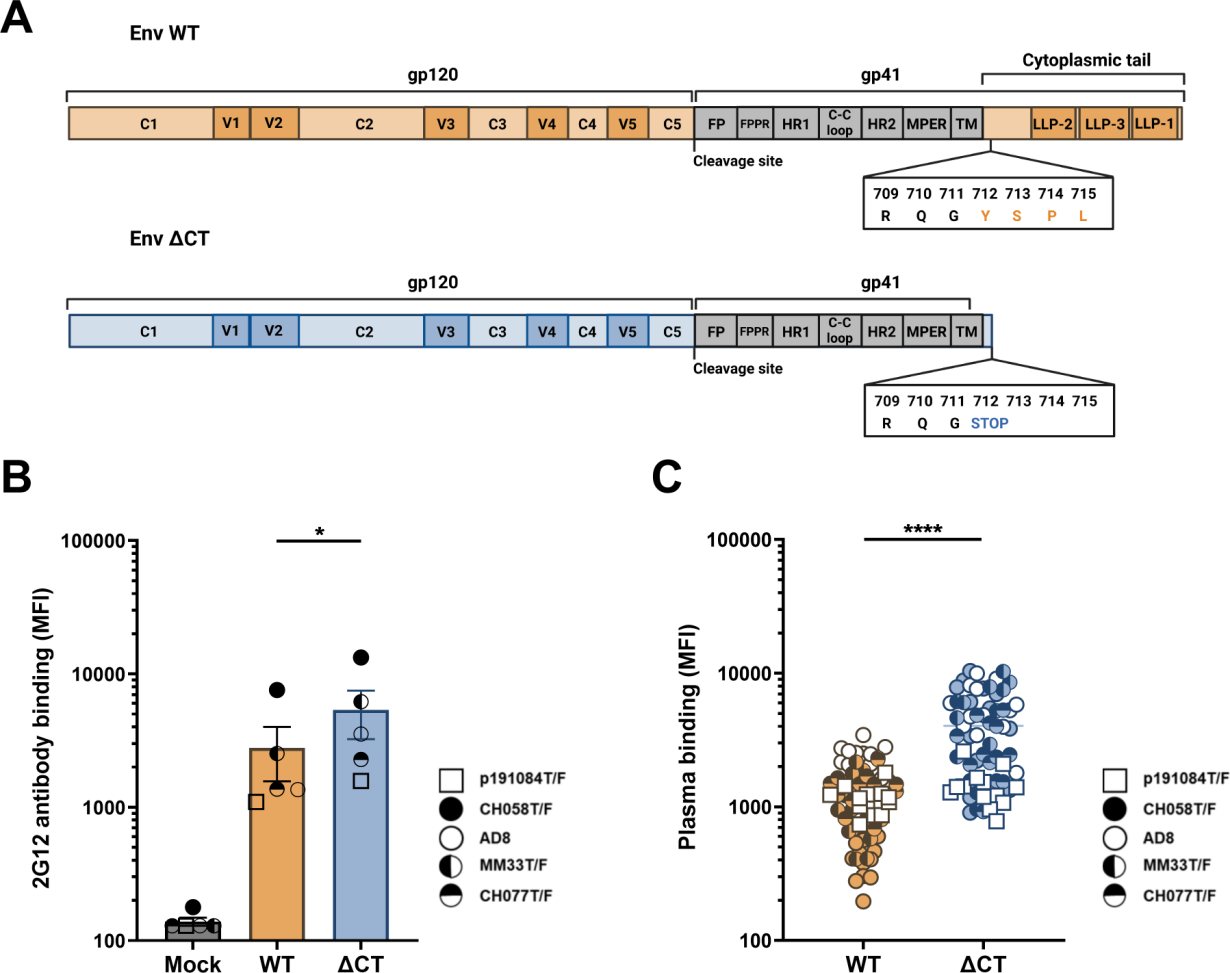
The HIV-1 Env cytoplasmic tail protects infected cells from detection by plasma from people living with HIV-1. (**A**) Schematic representation of the different domains of Env. C1–C5, constant domains; FP, fusion peptide; FPPR, fusion peptide proximal region; HR, heptad repeat; LLP, lentivirus lytic peptides; MPER, membrane proximal external region; TM, transmembrane domain; V1–V5, variable loops. (**B, C**) Primary CD4^+^ T cells were mock-infected or infected with viruses expressing either wild-type (WT) or cytoplasmic tail-truncated (∆CT) Env from different clades. Two days post-infection, the cells were stained with (**B**) the conformation-independent 2G12 monoclonal antibody (every symbol represents the mean of 2G12 binding for each IMC) or (**C**) 14 plasmas from PLWH. Shown are the median fluorescence intensities (MFI). Error bars indicate means ± SEM (*P < 0.05, ****P < 0.0001). Statistical significance was tested using paired *t*-tests, based on statistical normality.

Env gets incorporated into virions through interaction of its cytoplasmic tail with the viral matrix (MA) protein (12), but other functions of this intracellular Env domain in viral replication are less understood (13). The Env cytoplasmic tail contains a membrane-proximal YxxΦ endocytosis motif responsible for Env internalization and recycling by the AP-2 clathrin-dependent pathway (Fig. 1A) (14). Mutations in this motif increase Env levels at the surface of infected cells, resulting in enhanced susceptibility to ADCC mediated by purified IgG from PLWH (15). However, it was previously shown that enhanced levels of Env at the surface of infected cells are not sufficient to render them more susceptible to ADCC by CD4i antibodies or plasma from PLWH (16). Instead, the transition of Env to a more “open” conformation, similar to the one induced by CD4 interaction, was also required (16). Therefore, we investigated whether the cytoplasmic tail of Env contributed to HIV-1-mediated CD4 downregulation to prevent this conformational change.

We inserted a stop codon at position Y712 of the cytoplasmic tail, thereby truncating the tail just before the YxxΦ motif, in nine infectious molecular clones (IMCs) from four different clades (Fig. 1A). IMCs expressing either wild-type (WT) or cytoplasmic tail-deleted (∆CT) Env were used to infect primary CD4^+^ T cells isolated from human peripheral blood mononuclear cells (PBMCs) from six HIV-negative donors. We first measured Env levels at the surface of infected cells using the 2G12 antibody, which recognizes an epitope that is unaffected by changes in Env conformation (2, 17). As expected, we observed enhanced Env levels at the surface of Env ∆CT HIV-1-infected cells compared to their WT counterparts (Fig. 1B; Fig. S1A). Deletion of the cytoplasmic tail also significantly improved Env recognition by plasma from PLWH (Table S1; Fig. 1C; Fig. S1B), thus suggesting that Env is present in a more “open” conformation upon truncation of its cytoplasmic tail.

**Fig 2:**
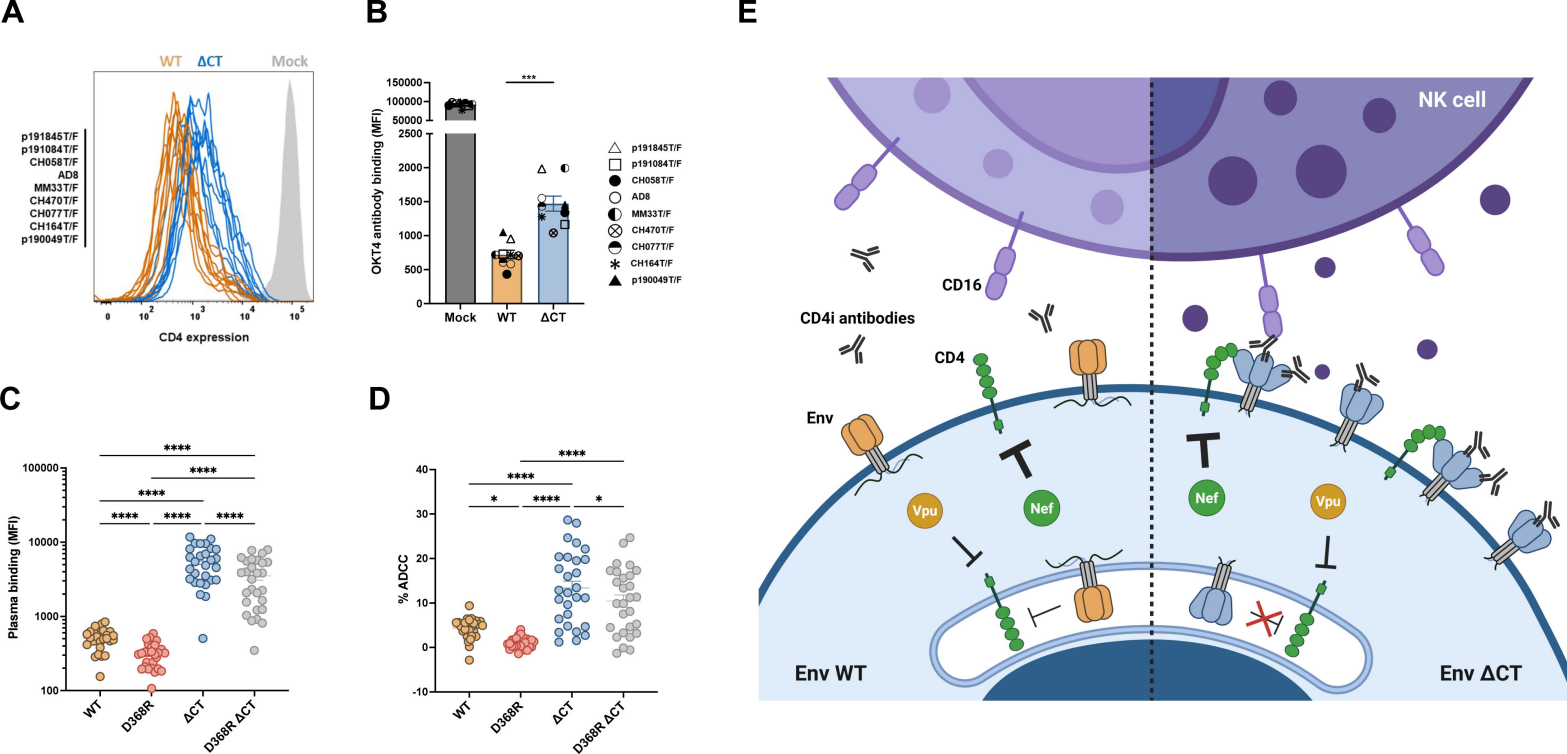
The HIV-1 Env cytoplasmic tail protects infected cells from ADCC by contributing to CD4 downregulation. (**A, B**) Primary CD4^+^ T cells were either mock-infected or infected with nine different IMCs from four different clades, each expressing either WT or ∆CT Env. Two days post-infection, the cells were stained with the anti-CD4 OKT4 antibody to measure cell-surface CD4 levels. (**A**) Representative histograms showing surface CD4 expression on CD4^+^ T cells, either mock-infected (gray) or infected with indicated IMCs expressing WT (orange) or ∆CT (blue) Env. (**B**) Every symbol represents the mean of anti-CD4 OKT4 binding obtained for each IMC variant in at least four independent experiments. (**C, D**) Primary CD4^+^ T cells were infected with the transmitted/founder virus HIV-1_CH058T/F_ expressing either the WT, D368R, ∆CT, or D368R ∆CT Env. Two days post-infection, (**C**) Env recognition and (**D**) ADCC-mediated elimination of infected cells by 28 plasmas from PLWH were measured by flow cytometry. Error bars indicate means ± SEM (*P < 0.05; ***P < 0.001; ****P < 0.0001). Shown are the MFI and percentage of ADCC obtained. Statistical significances were tested using (**B**) paired *t*-tests, (**C**) RM one-way ANOVA, and (**D**) Friedman test, based on statistical normality. (**E**) Overview of the effects of Env cytoplasmic tail truncation: increase in Env levels at the surface of infected cells; intrinsic “opening” of Env; increase in CD4 levels resulting in the exposure of otherwise occluded internal Env epitopes.

To evaluate whether this was the case, we probed these cells with four well-characterized CD4i antibodies recognizing the gp120 cluster A region (A32), the co-receptor binding site (17b), the V3 loop (19b), and the gp41 cluster I region (246D). In agreement with their CD4i nature, these antibodies poorly recognized cells infected with the WT virus but readily did so upon cytoplasmic tail truncation (Fig. S2A). Since CD4 expression at the cell surface is one of the main factors affecting Env conformation, we next measured CD4 levels using the OKT4 antibody. Remarkably, deletion of the Env cytoplasmic tail led to a small, but significant, accumulation of CD4 at the surface of cells infected with the nine IMCs tested (Fig. 2A and B; Fig. S3). These results indicated an important role of the Env cytoplasmic tail in CD4 downregulation.

To evaluate whether this small CD4 accumulation at the surface of infected cells contributed to Env recognition by plasma from PLWH and CD4i antibodies, we introduced a mutation in the gp120 CD4-binding site (CD4bs) at position D368 of HIV-1_CH058T/F_ expressing the WT and ∆CT Env. The D368R mutation abrogates Env-CD4 interaction, thereby keeping Env in its “closed” conformation (2), as well as the effect of the cytoplasmic tail on CD4 levels (Fig. S4). Supporting a role for Env-CD4 interaction in the enhanced recognition of infected cells by CD4i antibodies and plasma from PLWH, insertion of this mutation decreased Env recognition by these ligands (Fig. 2C; Fig. S2A). Additionally, recognition of the gp41 cluster I region by the 246D antibody was strongly decreased by insertion of the D368R mutation. Similarly, exposure of the gp120 cluster A region (recognized by A32) was abrogated by introduction of this mutation. Of note, the effect of the D368R mutation was less pronounced for the 17b and 19b antibodies that efficiently recognize the D368R ∆CT Env (Fig. S2A). These results are in agreement with previous studies showing that cytoplasmic tail deletions enable Env to sample downstream “open” conformations more readily, exposing epitopes normally occluded such as the co-receptor binding site or the V3 loop, even in the absence of CD4 (18).

We next measured ADCC mediated by plasma from PLWH (Fig. 2D) and CD4-induced antibodies (Fig. S2B) against HIV-1_CH058T/F_ expressing WT or ∆CT Env in the presence and absence of the D368R mutation. As expected, little ADCC was observed with WT Env (Fig. 2D). Cytoplasmic tail deletion increased ADCC mediated by plasma from PLWH and 17b, 246D, and 19b; however, this was not the case for A32, suggesting that the tail truncation-induced conformational change was not sufficient to mediate efficient binding by this antibody (Fig. 2D; Fig. S2B). Importantly, insertion of the D368R mutation decreased ADCC responses mediated by plasma from PLWH and CD4i antibodies.

In summary, our results support a model (Fig. 2E) where deletion of the cytoplasmic tail leads to a better recognition and elimination of HIV-1-infected cells by plasma from PLWH due to (i) an increase in Env levels at the surface of infected cells (15) as well as (ii) an increase in CD4 levels which results in the exposure of otherwise occluded internal trimer epitopes. Moreover, intrinsic “opening” of Env upon cytoplasmic tail truncation (18) also contributes to this phenotype. Additional work is required to determine how the cytoplasmic tail of Env contributes to CD4 downregulation.

## SUPPLEMENTAL MATERIAL

Supplemental material mbio.01763-25-s0001.pdf Supplemental figures and table.

Supplemental text mbio.01763-25-s0002.pdf Supplemental materials and methods.

ASM does not own the copyrights to Supplemental Material that may be linked to, or accessed through, an article. The authors have granted ASM a non-exclusive, world-wide license to publish the Supplemental Material files. Please contact the corresponding author directly for reuse.
