# Supplementary material for: The HIV-1 envelope cytoplasmic tail protects infected cells from ADCC by downregulating CD4
Source: mBio. 2025 Sep 8;16(10):e01763-25. doi: 10.1128/mbio.01763-25 (PMC12505979; doi:10.1128/mbio.01763-25)
Supplement: Supplemental material — Supplemental figures and table. [file mbio.01763-25-s0001.pdf]

Table S1: Cohort of people living with HIV.

Group	Sex		Age (years)	Years since ART initiation	Viral load (copies/mL)	CD4 count (cells/mm^3)
	Female (n)	Male (n)				
ART-treated	3	18	51 (27-70)	15.8 (0.1-23)	Undetectable	699 (197-1250)
Untreated	0	7	33 (29-51)	N/A	6641 (132-372071)	452 (263-570)

*Values displayed are medians, with ranges in parentheses.

ART: antiretroviral therapy.

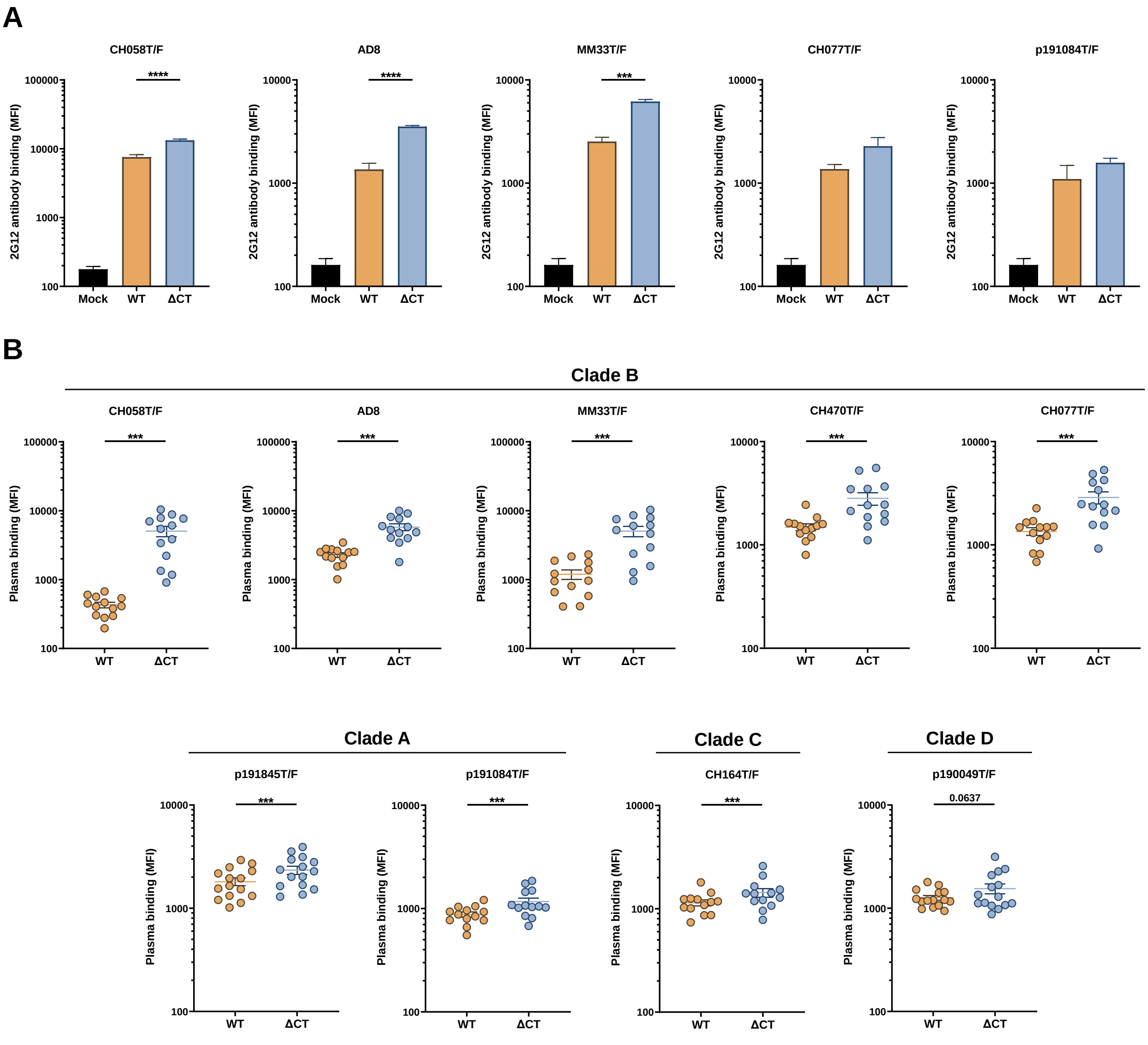

Figure S1. Env levels and recognition by plasma from PLWH at the surface of CD4⁺ T cells infected with WT or Env ΔCT viruses. Primary CD4⁺ T cells were mock-infected or infected with (**A**) 5 or (**B**) 9 primary viruses expressing either WT or ΔCT Env from (**A**) 2 or (**B**) 4 different clades. Two days post-infection, the cells were stained with (**A**) the conformation independent 2G12 antibody to measure Env levels at the surface of infected cells or (**B**) 14 plasma from PLWH. (**A**) The data shows the mean of at least 3 independent experiments. Error bars indicate means ± SEM (∗∗∗$p < 0.001$; ∗∗∗∗$p < 0.0001$). Statistical significance was tested using paired t tests or Wilcoxon matched-pairs signed rank test, based on statistical normality.

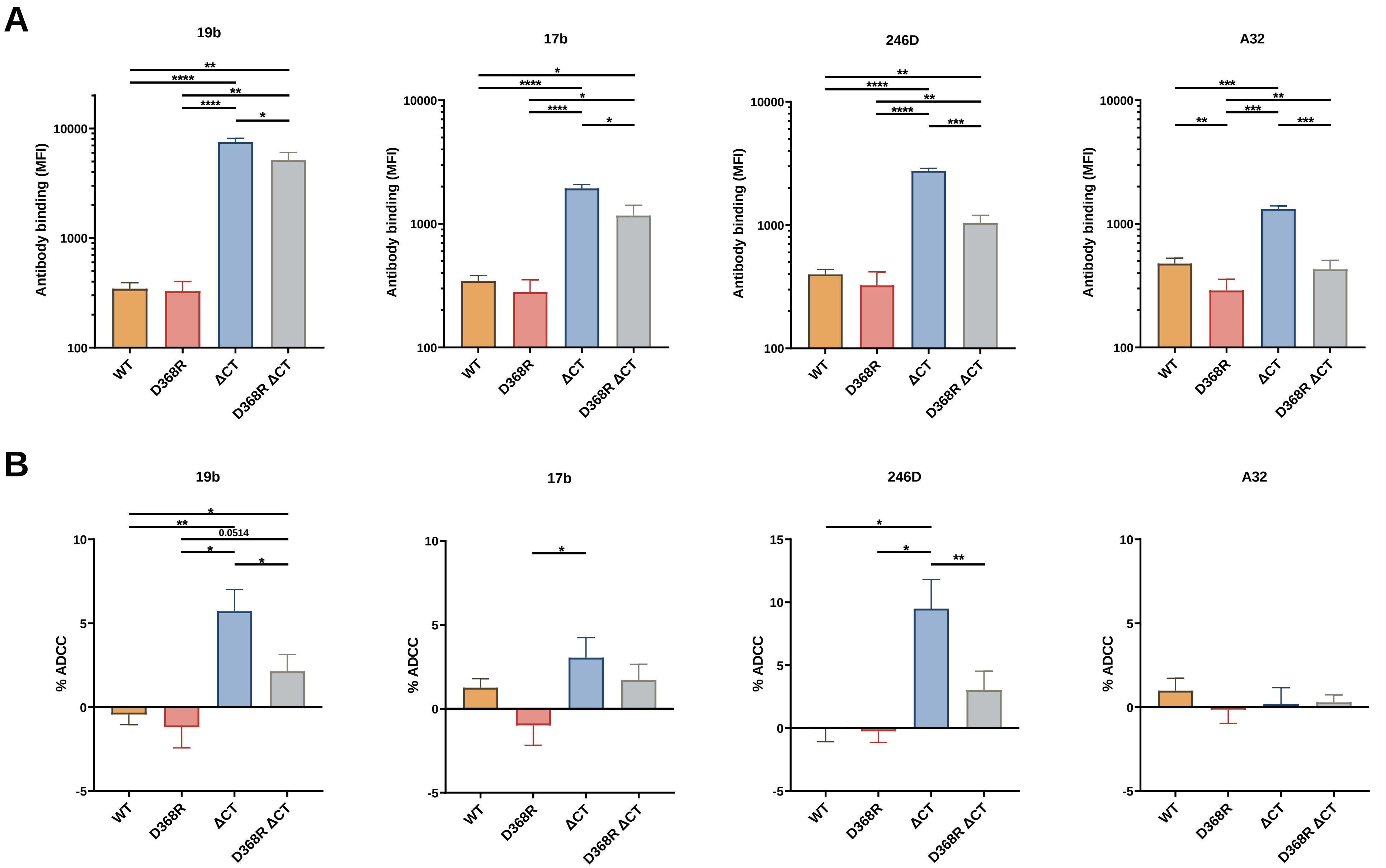

Figure S2. Deletion of Env cytoplasmic tail increases Env recognition and ADCC mediated by CD4-induced antibodies. (**A-B**) Primary CD4+ T cells were infected with the HIV-1$_{CH058T/F}$ expressing either WT, D368R, ΔCT or D368R ΔCT Env. Two days post-infection, (**A**) Env recognition and (**B**) ADCC activity mediated by 19b, 17b, 246D or A32 antibodies were measured by cell surface staining and ADCC assay respectively. The data shows the mean of 6 independent experiments. Error bars indicate means ± SEM ($*p < 0.05$; $**p < 0.01$; $***p < 0.001$; $****p < 0.0001$). Statistical significance was tested using paired t tests, based on statistical normality.

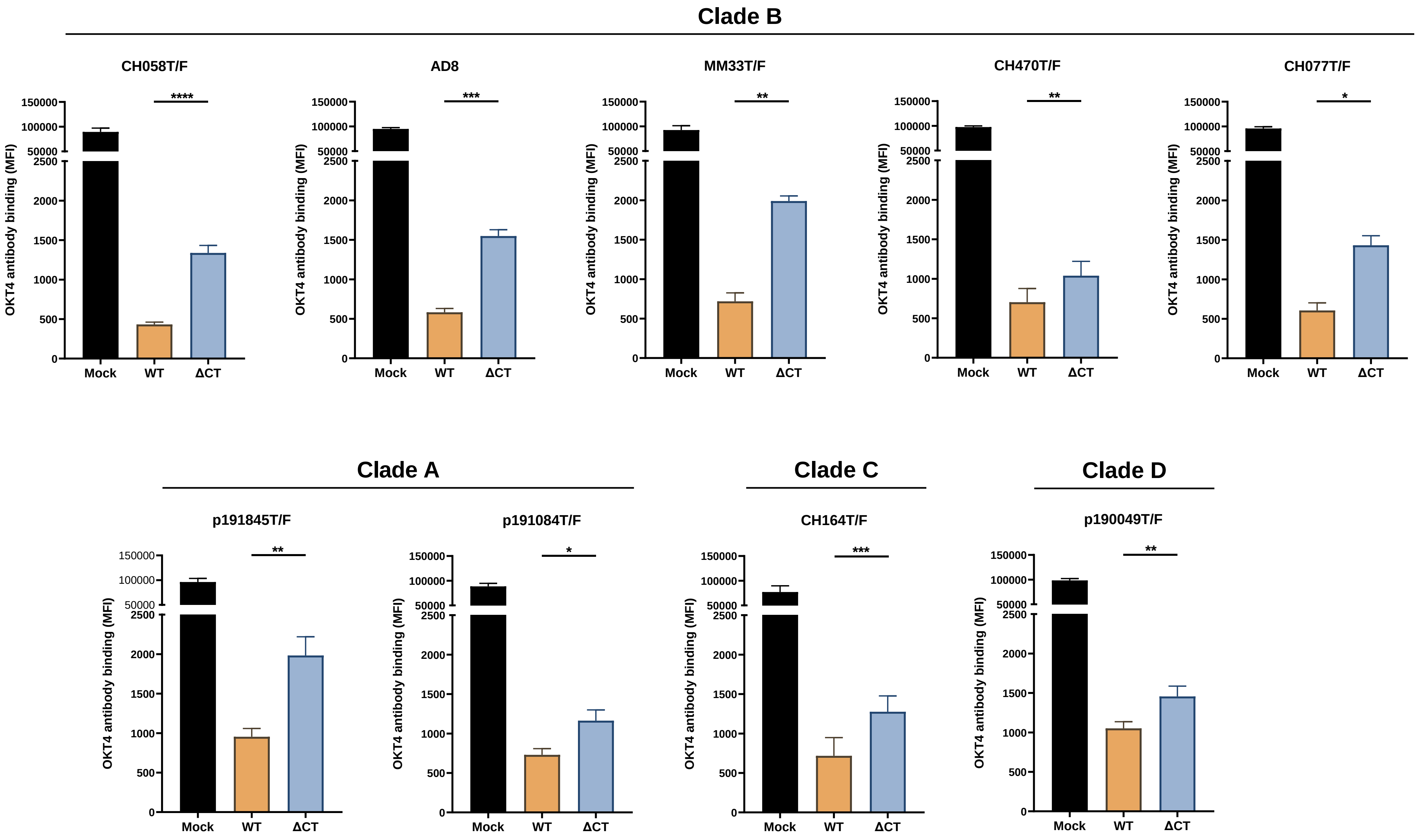

Figure S3. CD4 levels at the surface of CD4⁺ T cells infected with WT or Env ΔCT viruses. Primary CD4⁺ T cells were mock-infected or infected with 9 primary viruses expressing either WT or ΔCT Env from 4 different clades. Two days post-infection, the cells were stained with the anti-CD4 OKT4 antibody to measure CD4 levels at the surface of infected cells. The data shows the mean of at least 4 independent experiments. Error bars indicate means ± SEM ($*p < 0.05$; $**p < 0.01$; $***p < 0.001$; $****p < 0.0001$). Statistical significance was tested using paired t tests or Wilcoxon matched-pairs signed rank test, based on statistical normality.

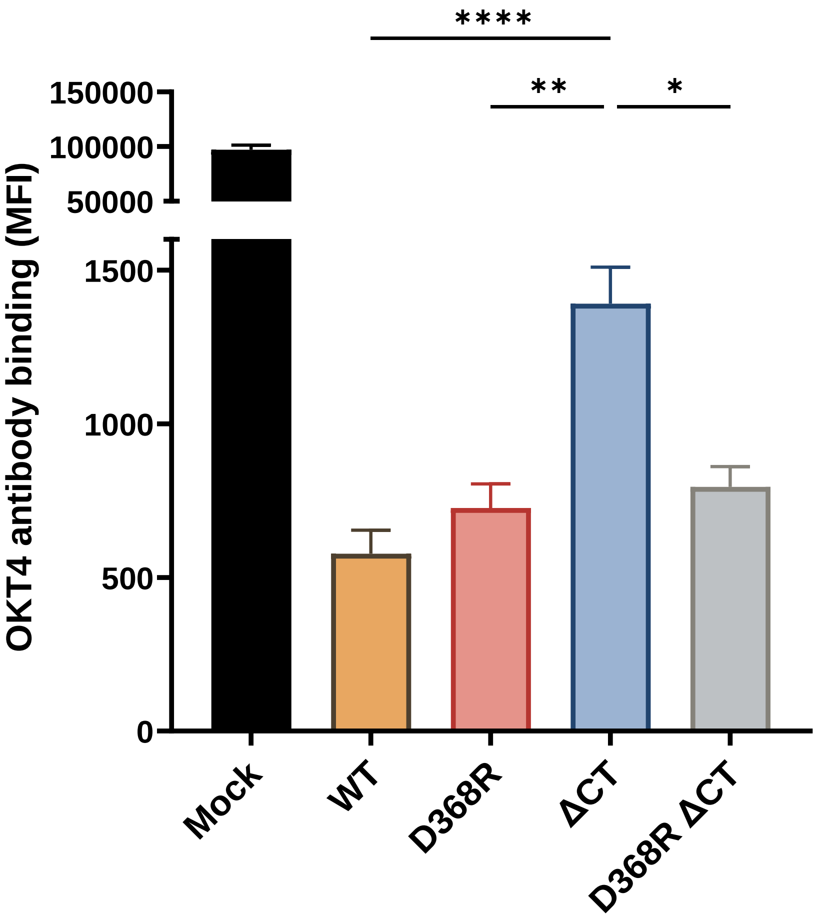

Figure S4. CD4 levels at the surface of CD4⁺ T cells infected with WT or Env ΔCT viruses harboring or not the D368R mutation. Primary CD4⁺ T cells were infected with the HIV-1$_{CH58T/F}$ expressing either WT, D368R, ΔCT or D368R ΔCT Env. Two days post-infection, the cells were stained with the anti-CD4 OKT4 antibody to measure CD4 levels at the surface of infected cells. The data shows the mean of 10 independent experiments. Error bars indicate means ± SEM (*$p < 0.05$; **$p < 0.01$; ****$p < 0.0001$). Statistical significance was tested using Friedman test, based on statistical normality.

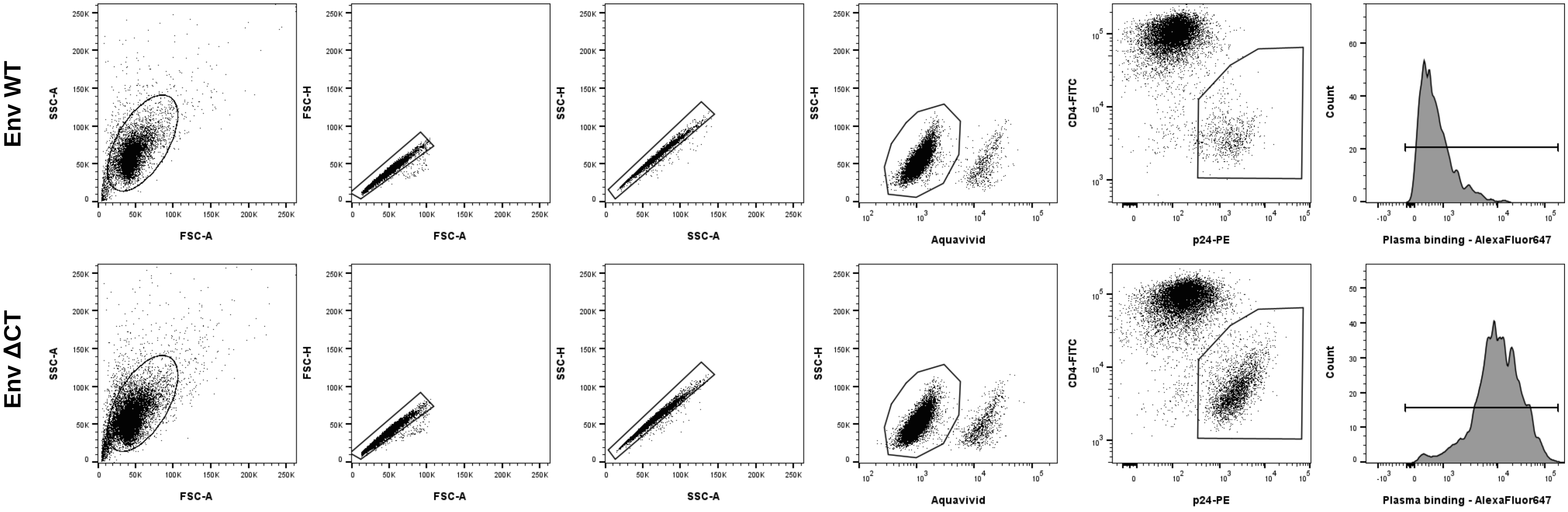

Figure S5. Gating strategy for cell surface staining. Representative flow cytometry gates to identify Env and CD4 levels at the surface of HIV-1 infected $CD4^+$ T cells (top: WT; bottom: ΔCT Env). HIV-1 infected $CD4^+$ T cells were stained with plasma or monoclonal antibodies and analyzed by flow cytometry. Cells were identified according to cell morphology by light-scatter parameters (first column) and excluding doublets cells (second and third columns). Cells were then gated on living cells (excluding the dead cells labeled with Aquavivid; fourth column). Finally, Env and CD4 binding by plasma or monoclonal antibodies was measured by the median of fluorescence of Alexa Fluor 647 (last column) in HIV-1 infected cells identified by gating on $p24^+CD4^{low}$ cells (fifth column).

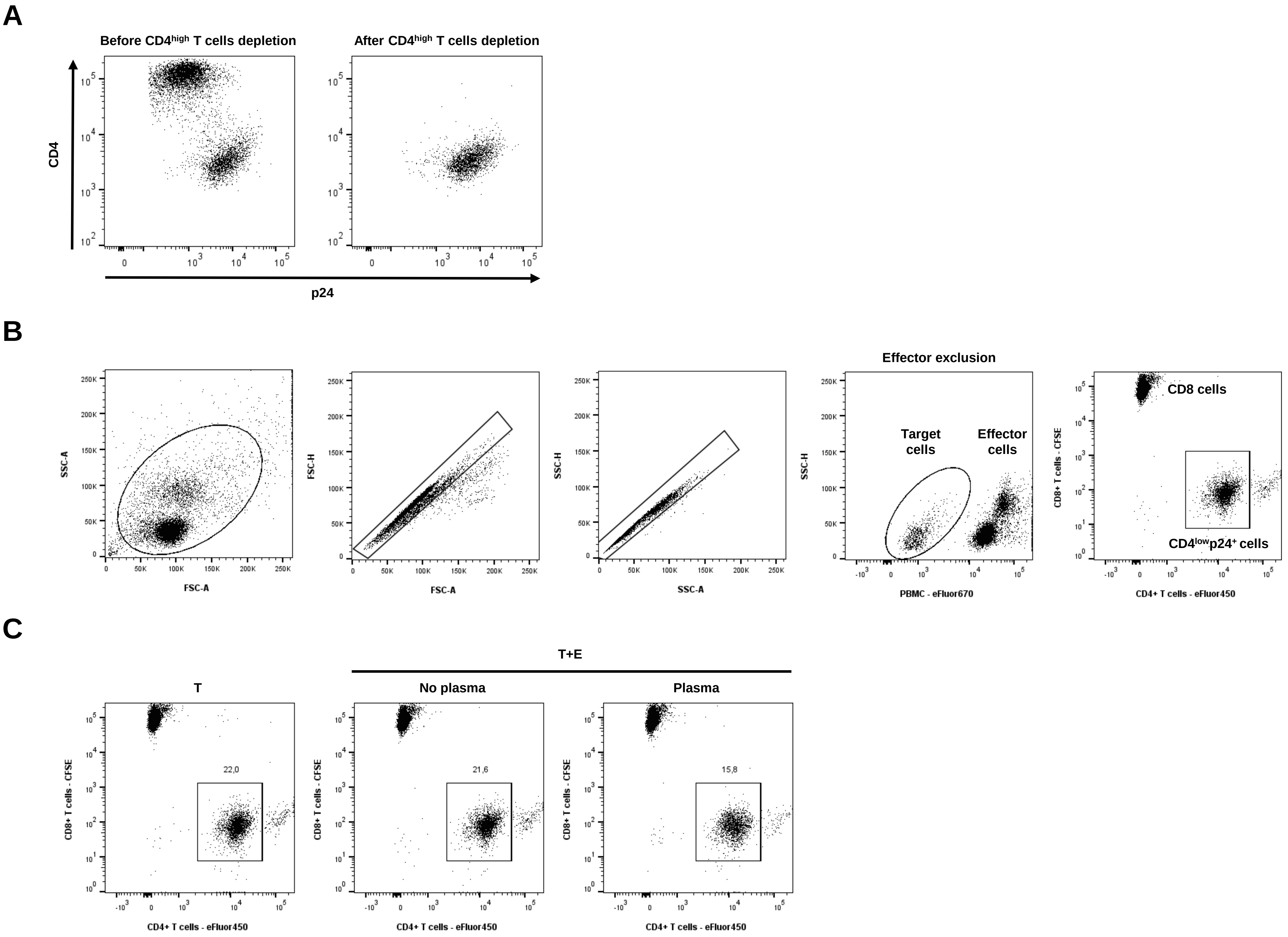

Figure S6. Gating strategies for ADCC assay. Primary CD4$^+$ T cells were infected with HIV-1$_{CH58T/F}$ expressing either WT, D368R, ΔCT or D368R ΔCT Env to perform ADCC assay. 48h post-infection, CD4^{high} uninfected bystander T cells coated with gp120 were depleted. (**A**) Representative flow cytometry gates to verify enrichement of productively-infected CD4^{low}p24$^+$ cells. The cells were stained with anti-CD4 OKT4 and anti-p24 monoclonal antibodies before and after removal of uninfected bystander CD4^{high} T cells. (**B**) Representative flow cytometry gates to measure ADCC activity. Total (target and effector cells) cells were identified according to cell morphology by light-scatter parameters (first column) and excluding doublets cells (second and third columns). Effectors cells were excluded by gating on eFluor670$^-$ cells (fourth column). Finally, CD4^{low}p24$^+$ target cells were identified by gating on eFluor450$^+$CFSE$^-$ cells (last column). (**C**) Representative plots for the eFluor450$^+$CFSE$^-$ gate for Targets alone (T), and Targets with Effectors (T+E) in presence or absence of plasma from PLWH.
