# Supplementary material for: The HIV-1 envelope cytoplasmic tail protects infected cells from ADCC by downregulating CD4
Source: mBio. 2025 Sep 8;16(10):e01763-25. doi: 10.1128/mbio.01763-25 (PMC12505979; doi:10.1128/mbio.01763-25)
Supplement: Supplemental text — Supplemental materials and methods. [file mbio.01763-25-s0002.pdf]

SUPPLEMENTAL MATERIALS AND METHODS

Supplemental material is available online.

Materials and methods have been previously reported in references (1–5) and are summarized below.

Ethics statement

Written informed consent was obtained from all study participants and research adhered to the ethical guidelines of CRCHUM and was reviewed and approved by the CRCHUM institutional review board (ethics committee, approval number MP-02-2024-11734 and 11.062). Research adhered to the standards indicated by the Declaration of Helsinki. All participants were adult and provided informed written consent prior to enrollment in accordance with Institutional Review Board approval.

Cell lines

HEK 293T human embryonic kidney cells (obtained from ATCC) were maintained in Dulbecco's Modified Eagle Medium (DMEM) (Wisent) supplemented with 5% fetal bovine serum (FBS) (VWR) and 100 U/mL penicillin/streptomycin (Wisent) at 37°C under 5% CO_2. FreeStyle 293F cells (Thermo Fisher Scientific) were grown in FreeStyle 293F medium (Thermo Fisher Scientific) to a density of 1×10^6 cells/mL at 37°C under 8% CO_2 with regular agitation (150 rpm).

Primary cells

Human peripheral blood mononuclear cells (PBMCs) from six HIV-negative individuals (four males and two females, age range 31-67 years) were obtained by leukapheresis and Ficoll density gradient isolation and were cryopreserved in liquid nitrogen until further use. $CD8^+$ T cells, used as control target cells in the ADCC assay, were isolated by negative selection following manufacturer's instruction (StemCell Technologies, Cat #17953) and cultured at 2 million of cells/mL overnight in RPMI 1640 (Thermo Fisher Scientific) complete medium supplemented with 20% FBS and 100 U/mL penicillin/streptomycin. $CD4^+$ T cells were purified from resting PBMCs by negative selection using immunomagnetic beads per the manufacturer's instructions (StemCell Technologies, Cat #17952), were activated with phytohemagglutinin-L (PHA-L, 10 µg/mL) for 48h and then maintained in RPMI 1640 complete medium supplemented with 20% FBS, 100 U/mL

penicillin/streptomycin and recombinant IL-2 (rIL-2, 100 U/mL). The cells were maintained at 37°C under 5% CO_2.

Antibodies production and purification

FreeStyle 293F cells were transfected with plasmids expressing the light (LC) and heavy (HC) chains of anti-Env monoclonal antibodies using ExpiFectamine 293 transfection reagent, as directed by the manufacturer (Thermo Fisher Scientific). One week later, the cells were pelleted and discarded. The supernatants were filtered (0.22-µm-pore-size filter), and the antibodies were purified by protein A affinity columns, as directed by the manufacturer (Cytiva). The recombinant protein preparations were dialyzed against phosphate-buffered saline (PBS) and stored in aliquots at -80°C. To assess purity, recombinant proteins were run on SDS-PAGE in the presence or absence of β-mercaptoethanol and stained with Coomassie blue.

Antibodies and plasmas

Plasma from PLWH, obtained from the FRQS-AIDS and Infectious Diseases Network (the Montreal Primary HIV Infection Cohort, Table S1)(6, 7), were collected, heat-inactivated for 1 h at 56°C and stored at −80°C until ready to use in subsequent experiments. Plasma were used at a 1:1000 dilution in cell surface staining and ADCC assays.

The following monoclonal antibodies were used at 5 ug/mL to detect Env expression at the cell surface of HIV-1-infected cells: anti-gp120 outer domain 2G12 antibody (plasmids for HC and LC obtained from the NIH AIDS Reagent Program); anti-cluster A A32 antibody (plasmids for HC and LC kindly provided by James Robinson); anti-CoRBS 17b antibody (plasmids for HC and LC kindly provided by James Robinson); anti-gp41 cluster I 246D antibody (plasmids for HC and LC obtained from the NIH AIDS Reagent Program); anti-V3 loop 19b antibody (plasmids for HC and LC obtained from the NIH AIDS Reagent Program). The anti-CD4 monoclonal antibody (Clone OKT4, eBioscience, Catalog #14-0048-82) was used to detect CD4 expression in cell surface staining (1 ug/mL). Alexa Fluor-647-conjugated goat anti-human IgG (Thermo Fisher Scientific, Cat #A-21445) was used as secondary antibody to detect anti-Env antibodies and plasma binding by flow cytometry (2 µg/mL). Alexa Fluor-647-conjugated goat anti-mouse IgG (Thermo Fisher Scientific, Cat # A-21235) was used as secondary antibody to detect mouse anti-human CD4 monoclonal antibodies binding by flow cytometry (2 ug/mL). The FITC anti-human CD4 antibody (Clone OKT4, Biolegend, Cat # 6604667) (1:500 dilution) and the PE-

conjugated anti-HIV p24 antibody (Clone KC57-RD1, Beckman Coulter, Cat # 6604667) (1:100 dilution) were used to identify productively-infected cells as previously described (8) in Env cell-surface staining.

Plasmids and proviral constructs

The vesicular stomatitis virus G (VSV-G)-encoding plasmid was previously described (7). The infectious molecular clones (IMCs) of HIV-1_{AD8} as well as the transmitted/founder viruses HIV-$1_{CH058T/F}$, HIV-$1_{CH470T/F}$, HIV-$1_{CH077T/F}$, HIV-$1_{CH164T/F}$, HIV-$1_{MM33T/F}$, HIV-$1_{p191845T/F}$, HIV-$1_{p191084T/F}$, HIV-$1_{p190049T/F}$ were previously described (10–15). The IMCs expressing ΔCT Env were generated by inserting a STOP codon in the YxxΦ internalization motif (at position Y712, according to HxBc2 numbering) by site-directed mutagenesis using the QuikChange II XL site-directed mutagenesis protocol (Agilent, Cat # 200521). The IMC HIV-$1_{CH058T/F}$ expressing D368R and D368R ΔCT Env were generated by inserting the D368R mutation into HIV-$1_{CH058T/F}$ expressing WT or ΔCT Env. The presence of the desired mutations was verified by automated DNA sequencing.

Viral production *and in vitro* infections

VSV-G-pseudotyped HIV-1 viruses were produced by co-transfection of HEK 293T cells with the HIV-1 IMC proviral constructs and the VSV-G-encoding vector at a ratio of 3:2 using the polyethylenimine (PEI) method. Two days post-transfection, cell supernatants were harvested, clarified by low-speed centrifugation (300 × g for 5 min), and concentrated by ultracentrifugation at 4°C (100,605 × g for 1h) over a 20% sucrose cushion. Pellets were resuspended in fresh RPMI 1640 complete medium, aliquoted and stored at -80°C until use. VSV-G-pseudotyped HIV-1 viruses were then used for *in vitro* infection. Activated primary $CD4^+$ T cells were spinoculated with the virus at 800 × *g* for 1h in 96-well plates at 25°C and then incubated 48h at 37°C. All viral productions were titrated on primary $CD4^+$ T cells to achieve similar levels of infection (around 12-20% of infected cells).

Flow cytometry analysis of cell-surface staining

Forty-eight hours post-infection, mock-infected and HIV-1-infected primary $CD4^+$ T cells were collected, washed with PBS and transferred in 96-well V-bottom plates. The cells were then incubated for 45 min at 37°C with plasma (1:1000 dilution) or the 2G12, 19b, 17b, 246D or A32 monoclonal antibodies (5 µg/mL) or anti-CD4 OKT4 antibody (1 ug/mL).

Cells were then washed twice with PBS and stained with appropriated anti-IgG Alexa Fluor 647-conjugated secondary antibody (2 μg/mL), FITC-conjugated mouse anti-human CD4 Antibody (Biolegend, 1:500 dilution) and LIVE/DEAD viability dye (Thermo Fisher Scientific, Cat #L34957, 1:1000 dilution) for 20 minutes at room temperature. Cells were then washed twice with PBS and fixed in a 2% PBS-formaldehyde solution. The cells were then permeabilized using the Cytofix/Cytoperm Fixation/Permeabilization Kit (BD Biosciences, Cat #554714) and stained intracellularly using PE-conjugated mouse anti-p24 monoclonal antibody (clone KC57, 1:100 dilution). Samples were acquired on a Fortessa cytometer (BD Biosciences), and data analysis was performed using FlowJo v10.5.3. Env and CD4 levels at the surface of infected cells were measured by the median of fluorescence of Alexa Fluor 647 in productively infected cells determined by gating on living $p24^{+}$ $CD4^{low}$ cell population as previously reported (8) (Figure S5).

ADCC assay

ADCC activity was measured 48h post-infection using a modified FACS-based infected cell elimination assay. It has been well established that soluble gp120 sheds from productively-infected cells and coats uninfected bystander cells; this substantially affects ADCC readings (4).To minimize this confounding factor and a potential effect of cytoplasmic tail truncation on gp120 shedding, the $CD4^{high}$ T cells, which represent the uninfected bystander cells coated with gp120, were depleted from the target cell population using the Dynabeads® $CD4^{+}$ positive selection kit (Thermo Fisher Scientific, Cat #11145D) at a ratio of 25 μl of beads per million cells as previously reported (3, 5). This procedure enriched the productively-infected $CD4^{low}p24^{+}$, as shown in Figure S6A.

To specifically assess ADCC-mediated killing of enriched productively-infected $CD4^{low}p24^{+}$ cells, autologous resting $CD8^{+}$ T cells, which are not susceptible to gp120 coating and ADCC, were included as control target cells (Figure S6B). The productively-infected $CD4^{low}p24^{+}$ cells were stained with the cell proliferation dye eFluor450 (Thermo Fisher Scientific, cat # 65-0842-90) while resting autologous $CD8^{+}$ T cells were stained with the cell proliferation dye CFSE (Thermo Fisher Scientific, cat # C34554). The $CD4^{low}p24^{+}$ cells were then mixed with $CD8^{+}$ T cells at a 1:3 ratio. Resting autologous PBMCs stained with the cell proliferation dye eFluor670 (Thermo Fisher Scientific, cat # 65-0840-90) were used as effectors cells. The target cell mix ($CD4^{low}p24^{+}$ cells and $CD8^{+}$ T cells) was then co-cultured with autologous PBMCs (Effector: Target ratio of 10:1) in 96-well V-bottom plates in the presence of plasma from PLWH (dilution 1:1000) or the 19b,

17b, 246D or A32 monoclonal antibodies (5µg/mL) for 5h at 37°C. After incubation, cells were washed once with PBS and fixed in a 2% PBS-formaldehyde solution. Samples were acquired on a Fortessa cytometer (BD Biosciences), and data analysis was performed using FlowJo v10.5.3. The percentage of ADCC was calculated as follows: [(% of eFluor450$^+$ cells in Targets plus Effectors) − (% of eFluor450$^+$ cells in Targets plus Effectors plus plasma)/(% of eFluor450$^+$ cells in Targets) × 100] (Figure 6C).

QUANTIFICATION AND STATISTICAL ANALYSIS

Statistics were analyzed using GraphPad Prism version 10.2.0. Every data set was tested for statistical normality and this information was used to apply the appropriate (parametric or nonparametric) statistical test. Statistical details of experiments are indicated in the figure legends. p values < 0.05 were considered significant; significance values are indicated as *p < 0.05, **p < 0.01, ***p < 0.001, ****p < 0.0001.

REFERENCES

1. Veillette M, Coutu M, Richard J, Batraville L-A, Dagher O, Bernard N, Tremblay C, Kaufmann DE, Roger M, Finzi A. 2015. The HIV-1 gp120 CD4-bound conformation is preferentially targeted by antibody-dependent cellular cytotoxicity-mediating antibodies in sera from HIV-1-infected individuals. J Virol 89:545–551.

2. Laumaea A, Marchitto L, Ding S, Beaudoin-Bussières G, Prévost J, Gasser R, Chatterjee D, Gendron-Lepage G, Medjahed H, Chen H-C, Smith AB, Ding H, Kappes JC, Hahn BH, Kirchhoff F, Richard J, Duerr R, Finzi A. 2023. Small CD4 mimetics sensitize HIV-1-infected macrophages to antibody-dependent cellular cytotoxicity. Cell Rep 42:111983.

3. Richard J, Prévost J, Baxter AE, Von Bredow B, Ding S, Medjahed H, Delgado GG, Brassard N, Stürzel CM, Kirchhoff F, Hahn BH, Parsons MS, Kaufmann DE, Evans DT, Finzi A. 2018. Uninfected Bystander Cells Impact the Measurement of HIV-Specific Antibody-Dependent Cellular Cytotoxicity Responses. mBio 9:e00358-18.

4. Richard J, Veillette M, Ding S, Zoubchenok D, Alsahafi N, Coutu M, Brassard N, Park J, Courter JR, Melillo B, Smith AB, Shaw GM, Hahn BH, Sodroski J, Kaufmann DE, Finzi A. 2016. Small CD4 Mimetics Prevent HIV-1 Uninfected Bystander CD4 + T Cell Killing Mediated by Antibody-dependent Cell-mediated Cytotoxicity. EBioMedicine 3:122–134.

5. Prévost J, Richard J, Ding S, Pacheco B, Charlebois R, Hahn BH, Kaufmann DE, Finzi A. 2018. Envelope glycoproteins sampling states 2/3 are susceptible to ADCC by sera from HIV-1-infected individuals. Virology 515:38–45.

6. Fontaine J, Coutlée F, Tremblay C, Routy J-P, Poudrier J, Roger M, Montreal Primary HIV Infection and Long-Term Nonprogressor Study Groups. 2009. HIV infection affects blood myeloid dendritic cells after successful therapy and despite nonprogressing clinical disease. J Infect Dis 199:1007–1018.

7. Fontaine J, Chagnon-Choquet J, Valcke HS, Poudrier J, Roger M, Montreal Primary HIV Infection and Long-Term Non-Progressor Study Groups. 2011. High expression levels of B lymphocyte stimulator (BLyS) by dendritic cells correlate with HIV-related B-cell disease progression in humans. Blood 117:145–155.

8. Richard J, Sannier G, Zhu L, Prévost J, Marchitto L, Benlarbi M, Beaudoin-Bussières G, Sun Y, Hongil K, Chatterjee D, Medjahed H, Bourassa C, Delgado G-G, Dubé M, Kirchhoff F, Hahn BH, Kumar P, Kaufmann DE, Finzi A. 2024. CD4 downregulation precedes Env expression and protects HIV-1-infected cells from ADCC mediated by non-neutralizing antibodies. bioRxiv https://doi.org/10.1101/2024.05.01.592003.

9. Emi N, Friedmann T, Yee JK. 1991. Pseudotype formation of murine leukemia virus with the G protein of vesicular stomatitis virus. J Virol 65:1202–1207.

10. Ochsenbauer C, Edmonds TG, Ding H, Keele BF, Decker J, Salazar MG, Salazar-Gonzalez JF, Shattock R, Haynes BF, Shaw GM, Hahn BH, Kappes JC. 2012. Generation of Transmitted/Founder HIV-1 Infectious Molecular Clones and Characterization of Their Replication Capacity in CD4 T Lymphocytes and Monocyte-Derived Macrophages. J Virol 86:2715–2728.

11. Theodore TS, Englund G, Buckler-White A, Buckler CE, Martin MA, Peden KWC. 1996. **Short Communication** : Construction and Characterization of a Stable Full-

Length Macrophage-Tropic HIV Type 1 Molecular Clone That Directs the Production of High Titers of Progeny Virions. AIDS Res Hum Retroviruses 12:191–194.

12. Gondim MVP, Sherrill-Mix S, Bibollet-Ruche F, Russell RM, Trimboli S, Smith AG, Li Y, Liu W, Avitto AN, DeVoto JC, Connell J, Fenton-May AE, Pellegrino P, Williams I, Papasavvas E, Lorenzi JCC, Salantes DB, Mampe F, Monroy MA, Cohen YZ, Heath S, Saag MS, Montaner LJ, Collman RG, Siliciano JM, Siliciano RF, Plenderleith LJ, Sharp PM, Caskey M, Nussenzweig MC, Shaw GM, Borrow P, Bar KJ, Hahn BH. 2021. Heightened resistance to host type 1 interferons characterizes HIV-1 at transmission and after antiretroviral therapy interruption. Sci Transl Med 13.

13. Sugrue E, Wickenhagen A, Mollentze N, Aziz MA, Sreenu VB, Truxa S, Tong L, Da Silva Filipe A, Robertson DL, Hughes J, Rihn SJ, Wilson SJ. 2022. The apparent interferon resistance of transmitted HIV-1 is possibly a consequence of enhanced replicative fitness. PLOS Pathog 18:e1010973.

14. Koyanagi Y, Miles S, Mitsuyasu RT, Merrill JE, Vinters HV, Chen ISY. 1987. Dual Infection of the Central Nervous System by AIDS Viruses with Distinct Cellular Tropisms. Science 236:819–822.

15. Baalwa J, Wang S, Parrish NF, Decker JM, Keele BF, Learn GH, Yue L, Ruzagira E, Ssemwanga D, Kamali A, Amornkul PN, Price MA, Kappes JC, Karita E, Kaleebu P, Sanders E, Gilmour J, Allen S, Hunter E, Montefiori DC, Haynes BF, Cormier E, Hahn BH, Shaw GM. 2013. Molecular identification, cloning and characterization of transmitted/founder HIV-1 subtype A, D and A/D infectious molecular clones. Virology 436:33–48.
